# A two-stage approach to the joint analysis of longitudinal and survival data utilising the Coxian phase-type distribution

**DOI:** 10.1177/0962280217706727

**Published:** 2017-06-20

**Authors:** Conor Donnelly, Lisa M McFetridge, Adele H Marshall, Hannah J Mitchell

**Affiliations:** Mathematical Sciences Research Cluster, Queen’s University Belfast, Northern Ireland, UK

**Keywords:** Linear mixed effects models, Coxian phase-type distribution, longitudinal analysis, personalised medicine, Coxian regression model, haemoglobin, renal research

## Abstract

The Coxian phase-type distribution is a special type of Markov model which can be utilised both to uncover underlying stages of a survival process and to make inferences regarding the rates of flow of individuals through these latent stages before an event of interest occurs. Such models can be utilised, for example, to identify individuals who are likely to deteriorate faster through a series of disease states and thus require more aggressive medical intervention. Within this paper, a two-stage approach to the analysis of longitudinal and survival data is presented. In Stage 1, a linear mixed effects model is first used to represent how some longitudinal response of interest changes through time. Within this linear mixed effects model, the individuals’ random effects can be considered as a proxy measure for the effect of the individuals’ genetic profiles on the response of interest. In Stage 2, the Coxian phase-type distribution is employed to represent the survival process. The individuals’ random effects, estimated in Stage 1, are incorporated as covariates within the Coxian phase-type distribution so as to evaluate their effect on the individuals’ rates of flow through the system represented by the Coxian. The approach is illustrated using data collected on individuals suffering from chronic kidney disease, where focus is given to an emerging longitudinal biomarker of interest – an individual’s haemoglobin level.

## 1 Introduction

Personalised medicine has been described as ‘an emerging practice of medicine that uses an individual’s genetic profile to guide decisions made in regard to the prevention, diagnosis, and treatment of disease’.^1^ The aim is to identify and measure the effect of individual-specific characteristics, in addition to standard covariates, which may be indicative of how a disease will behave or of what impact a certain treatment will have on the rate of disease onset. In doing so, medical intervention can be tailored to suit an individual’s unique requirements, moving away from previous ‘one size fits all’ treatment approaches which often prove inefficient. Indeed, it is estimated that standard prescription drugs, currently on the market, work for only half of those who take them.^2^ The problem, however, is that it is difficult to obtain a good measure of an individual’s genetic profile so as to incorporate its effect within a statistical model.

Common approaches to personalised medicine focus, at least partially, on looking at various biomarkers which have a possible underlying association with the disease of interest. Such biomarker covariates, which change in a way that reflects the individual’s ‘internal’ health status, are referred to as endogenous covariates and they can serve as a proxy measure of an individual’s health condition. These endogenous covariates are assumed to be in some way influenced by the effects of both observable exogenous covariates as well as the individual’s unobserved genetic profile. It would be expected, then, to perceive distinct variation amongst the endogenous covariates across individuals; this is attributed to the uniqueness of each individual’s genetic profile. Indeed, no matter how many exogenous covariates are observed, it is never going to be possible to completely account for all the variation which exists between individuals.

Linear mixed effects (LME) models are a powerful statistical tool which can be employed to quantify the variation observed amongst individuals in situations where multiple repeated measures have been made on the individual’s covariate values over time.^3^ They do this by considering the response variable of interest to be influenced by both observed covariates, or ‘fixed effects’, and latent individual characteristics, that is, the effects of the underlying genetic profile represented by random effects. These random effects, which give a measure of how an individual varies from the overall population average, can then be utilised, for example, to determine how a disease is going to progress for a specific individual of interest or how a particular individual will likely respond to a certain drug. Accurate prediction from the conditional distribution of the random effects thus would allow treatment intervention to be determined in a personalised manner.

Previous research has shown LME models to be an effective approach to estimating individual-specific trajectories of some endogenous biomarker covariate. By subsequently incorporating the predicted random effects within a survival model, it is possible to evaluate the influence of an individual’s genetic profile on their survival. For example, Tsiatis et al.,^4^ Self and Pawitan^5^ and De Gruttola and Tu^6^ have each shown that the CD4 cell count trajectories of individuals suffering from HIV are a significant predictor of time until AIDS diagnosis. Furthermore, by incorporating the predicted random effects within a Cox proportional hazards (PH) model, they showed that it is possible to evaluate the extent of this effect. Similar research has been conducted to model the relationship between disease progression markers and survival outcome in a wide range of medical investigations, for instance, prostate cancer,^7^ schizophrenia^8^ and lung disease,^9^ to give just a few examples. Similarly, accelerated failure time models have also been utilised in such studies to represent the survival process when the PH assumption does not hold.^10^

Whilst the aforementioned approaches have proven useful in assessing the relationship between longitudinal response and event outcome, they do not provide much information regarding the quality of health an individual will experience before the event of interest is realised. Such insight could be utilised to further inform decision making. For instance, consider two individuals who suffer from the same disease and who have the same estimated survival time, say 10 years. It may be that the disease of interest has three underlying stages of progression: ‘Stage 1’, in which individuals are infected with the disease which remains dormant within the body, ‘Stage 2’ within which the disease remains asymptomatic but begins to attack the body’s immune system and ‘Stage 3’, in which physical symptoms of the disease begin to manifest, negatively affecting the individual’s health condition. An individual who spends one year in ‘Stage 1’, one year in ‘Stage 2’ and eight years in ‘Stage 3’, will have a much reduced quality of life, and require a different treatment plan, compared to an individual who will spend eight years in ‘Stage 1’, one year in ‘Stage 2’ and one year in ‘Stage 3’. Furthermore, the need to analyse disease progression is more imperative in cases where treating an infected individual with a particular drug intervention when the disease becomes active (i.e. when an individual transitions into Stage 2) decreases the rate of deterioration through the disease stages, extending the time for which the individual will remain in good health. Without knowing the rates of progression of individuals through these stages, however, it may be that some individuals are not prescribed the drug early enough to benefit from its full effects or, conversely, some individuals may be prescribed the drug too early which can be an unnecessary cost to either the individual or the healthcare provider.

Phase-type distributions are a useful statistical instrument which can be utilised in survival analysis to represent how an individual behaves before experiencing some event of interest.^11^ Conceptually, they consider the survival time to be broken down into a number of distinct states or phases, representing different stages of the survival process, through which individuals transition as their condition evolves. More formally, phase-type distributions, as described by Neuts, are a mathematically tractable way of modelling the time to absorption, *T*, of a continuous-time, finite Markov chain which begins in a transient state and ends in a single absorbing state.^12^

While phase-type distributions have enjoyed particular prominence in queueing theory,^13^ they have also proven useful in survival analysis studies where they have been used to successfully model the time until some event under investigation occurs. For example, Aalen^11^ discussed the extension of the phase-type paradigm from standard queueing theory to the arena of biostatistics, specifically modelling the incubation time of AIDS.

This paper explores the use of the Coxian phase-type distribution, a particular subclass of the general phase-type distribution within which the phases are ordered and only sequential transitions between them are permitted, representing the underlying stages of a disease. In particular, focus will be given to the Coxian phase-type regression model to enable the analysis of factors which impact survival.^14^ Specifically, the random effects of a LME model, fitted to a repeatedly observed, endogenous covariate which is associated with an event outcome of interest, are incorporated as predictors within the Coxian phase-type regression model. This approach not only allows inferences to be made regarding the effect of individuals’ repeated measures trajectories on survival time, similarly to current approaches in the literature, but extends this to also allow inferences to be made on the rates of deterioration through the various stages of the disease and subsequent movement into the absorbing state. Consequently, more accurate treatment plans can be designed, catering to individuals’ personal requirements, moving away from a single treatment plan for the entire population.

Additionally, this paper employs the survivor and hazard functions of the Coxian phase-type distribution to predict both population-average and individual-specific survival probabilities and hazards through time. These predictions are compared to the empirical plots, showing how such methods provide good fits to the data. This allows for more accurate predictions of survival probability and life expectancy to be made, catered to the individual’s profile.

The remainder of this paper is laid out as follows. Section 2 explores the LME model in more detail. In Section 3 the Coxian phase-type distribution is presented and the fitting procedure discussed. The Coxian phase-type regression model, capable of evaluating the effect of various covariates on transition rates through the system represented by the Coxian phase-type distribution, is reviewed in Section 4. Finally, Section 5 applies the new two-step approach to the analysis of individuals suffering from chronic kidney disease (CKD), with some conclusions discussed in Section 6.

## 2 LME models

LME models are a generalised approach to fit a linear regression to data whereby there are multiple clustered or correlated observations made on a single response, for example within a longitudinal study. They work by considering the response variable of interest (some periodically observed, endogenous covariate) to be influenced by both fixed effects and unobserved, individual-specific, random effects. Within this context, the fixed effects are the observed covariates within the model which are presumed to have a constant (or ‘fixed’) effect on the rate of change of the response variable across all individuals. The random effects, on the other hand, represent the characteristics of an individual which are unobserved within the model but which may still have an effect on the response variable.^3^ Provided that there are sufficient fixed effects within the model to represent the observable characteristics of the individuals, the random effects can be considered as a measure of the latent effect of a subject’s genetic profile on the endogenous covariates, which, themselves, serve as a proxy measure of the individual’s health condition.

In regard to personalised medicine, the potential to measure how one individual’s disease biomarkers, or other endogenous covariates, change over time, relative to the population-average change, offers a number of advantages. It may be that there exists some association between the dynamic nature of an individual’s deviation from the population average and some future, disease-related event outcome. This could be beneficial if, for example, observing the biomarker’s change on an individual level over time could be used to inform physicians of some potential future event so that an intervention can be implemented to prevent the negative outcome.

In matrix notation, the generalised LME model for the *i*th individual, introduced by Laird and Ware,^3^ is given by

(1) $$y_{i}=X_{i}\beta +Z_{i}b_{i}+{ɛ}_{i},i=1,\ldots ,M$$

where

$y_{i}$ is an $m_{i}\times 1$ vector of the *m_i_* observed responses for individual *i*,

$X_{i}$ is an $m_{i}\times l$ design matrix of the *l* observed explanatory variables for individual *i*,

β is an l×1 vector of the unknown population parameters (fixed effects),

$Z_{i}$ is an $m_{i}\times q$ design matrix of the *q* random effects for individual *i*,

$b_{i}$ is a q×1 vector of the latent, individual-specific random effects,

${ɛ}_{i}$ is an $m_{i}\times 1$ vector of the residual error terms.

It is assumed that the random effects follow a bivariate normal distribution with zero mean and covariance matrix **D** such that

(2) $$b_{i}=\left(\begin{array}{c} b_{i0}\\ b_{i1} \end{array}\right)$$

where $b_{i0}$ and $b_{i1}$ are measures of how individual *i*’s intercept and rate of change over time vary from the population average, respectively.

Similarly, it is assumed that the residual errors are normally distributed, ${ɛ}_{i}∼N(0,R_{i})$. Here, $R_{i}$ is an $m_{i}\times m_{i}$ positive-definite covariance matrix of the individual’s residual errors. It is commonly assumed that all individuals’ observations are independent and identically distributed random variables where $R_{i}$ is given by $\sigma^{2}I_{m_{i}}$.^3,15^ The unknown parameter, $\sigma^{2}$, is not individual specific but the dimension of $I_{m_{i}}$ (and thus the dimension of $\sigma^{2}I_{m_{i}}$) is as shown

(3) $$R_{i}=Var(\epsilon_{i})=\left[\begin{array}{cccc} \sigma^{2} & 0 & \;\ldots & 0\\ 0 & \sigma^{2} & \;\ldots & 0\\ \vdots & \vdots & \ddots & \vdots \\ 0 & 0 & \;\ldots & \sigma^{2} \end{array}\right]=\sigma^{2}\left[\begin{array}{cccc} 1 & 0 & \;\ldots & 0\\ 0 & 1 & \;\ldots & 0\\ \vdots & \vdots & \ddots & \vdots \\ 0 & 0 & \;\ldots & 1 \end{array}\right]=\sigma^{2}I_{m_{i}}$$

The unknown parameters of the LME model, Ψ = ($\beta ,\sigma^{2}$ and **D**), are estimated using the method of maximum likelihood where the likelihood function is given by

(4) $$L(\Psi ,y_{i})=\overset{n}{\underset{i=1}{\Pi }}f(y_{i};\Psi )$$

The random effects are predicted using an extension of the Gauss–Markov theorem for random effects, given the estimates Ψ and the data under investigation.

## 3 The Coxian phase-type distribution

### 3.1 Background

As already discussed, phase-type distributions represent the time to absorption of a continuous time, finite Markov chain which begins in a transient state and ends in a single absorbing state. Generally speaking, such distributions are formed by a convolution of exponentially distributed phases, either in series or parallel, constituting a combination of Poisson processes which, together, represent the overall time to absorption. The basis of this concept extends from Erlang’s^16^ ‘method of states’ where, in 1917, he used a series of identical exponential distributions to model telephone traffic. Since Erlang’s pioneering effort, phase-type distributions have been generalised in many ways and can be used to arbitrarily closely approximate any positive, continuous distribution through a system of *n* exponentially distributed phases.^17^

One of the advantages of phase-type distributions is that inferences can be made from the parameters of the distribution on the rates of flow through the underlying system of phases, where the parameters can be interpreted similarly to those of a multi-state Markov model. This is particularly beneficial when the underlying phases of the distribution map onto distinct stages of the survival process. In such cases, it is possible to gain insight into how individuals behave, in terms of their progression through the disease stages, before the event of interest is realised. For example, Faddy and McClean^17^ used phase-type distributions to analyse patient length of stay in hospital and found that the phases could be interpreted to represent increased severity of illness being treated and thus allowed them to identify short-stay, medium-stay and long-stay patients.

Despite the flexible nature of phase-type distributions, they do have some limitations. Primarily, they tend to be over-parameterised; a standard phase-type distribution has $\left(n^{2}+n\right)$ parameters, where *n* is the number of phases of the distribution, therefore making the fitting process difficult and computationally expensive. Commonly, therefore, a subclass of the general phase-type distribution, known as a Coxian phase-type distribution, is employed, which reduces the number of parameters to (2n-1) while still providing a suitable fit to the data.^18^

### 3.2 The Coxian phase-type distribution

**Figure 1.**
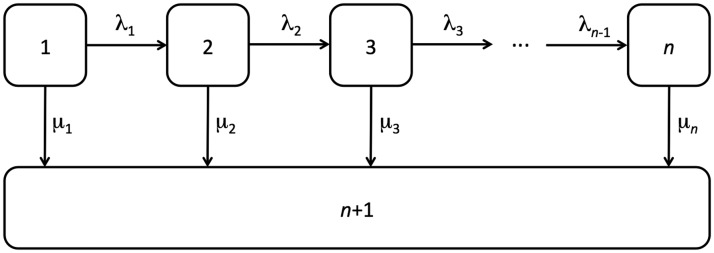
Diagram showing an *n*-phase Coxian phase-type distribution where the allowed transitions amongst the *n* phases are indicated by arrows, and where *λ_k_* represents the instantaneous risk of transitioning from phase *k* into phase *k* + 1 and *μ_k_* represents the instantaneous risk of transitioning from phase *k* into the absorbing phase, *n* + 1.

Conceptually, within the Coxian phase-type distribution, all individuals are considered to initially belong to the first phase of the system from which they can either move sequentially through the transient phases at rate *λ_k_*, or transition into the absorbing phase when the event of interest occurs at rate *μ_k_*. This underlying multistate structure represents the general flow of individuals diagnosed with a chronic or degenerative condition and is illustrated diagrammatically in Figure 1.

Mathematically, we can allow this latent Markov process to be defined by {X(t);t≥0}, with a state space {1,2,…,n+1}, where X(0)=1 and state *n* + 1 is the absorbing state. For k=1,…,n-1, the probability of a sequential transition amongst the transient states in an infinitesimal time interval *δt* is given by

(5) $$P\{X(t+\delta t)=k+1|X(t)=k\}=\lambda_{k}\delta t+o(\delta t)$$

where *λ_k_* represents the rates of movement sequentially through the transient phases. Similarly, for k=1,…,n, the probability of the event of interest occurring and of transition from any of the transient states into the absorbing phase is given by

(6) $$P\{X(t+\delta t)=n+1|X(t)=k\}=\mu_{k}\delta t+o(\delta t)$$

where *μ_k_* represents the rates of movement from the transient phases into the absorbing phase.^12^

The system can be represented in matrix notation where the probability density function of the Coxian phase-type distribution is given by

(7) f(t)=pexp{Qt}q

where **Q** is the phase-type generator matrix whose *ij*th entry (where i≠j) represents the instantaneous risk of transitioning from phase *i* to phase *j*

(8) $$Q=\left[\begin{array}{cccccc} -(\lambda_{1}+\mu_{1}) & \lambda_{1} & 0 & \;\ldots & 0 & 0\\ 0 & -(\lambda_{2}+\mu_{2}) & \lambda_{2} & \;\ldots & 0 & 0\\ 0 & 0 & -(\lambda_{3}+\mu_{3}) & \;\ldots & 0 & 0\\ \vdots & \vdots & \vdots & \ddots & \vdots & \vdots \\ 0 & 0 & 0 & \ldots & -(\lambda_{n-1}+\mu_{n-1}) & \lambda_{n-1}\\ 0 & 0 & 0 & \ldots & 0 & -\mu_{n} \end{array}\right]$$

**p** is a row vector of probabilities of initially belonging to each transient state

(9) $$p=\left(\begin{array}{cccccc} 1 & 0 & 0 & \ldots & 0 & 0 \end{array}\right)$$

and **q** is a vector of the rates of absorption from each transient state

(10) $$q=(\mu_{1}\mu_{2}\mu_{3}\ldots \mu_{n-1}\mu_{n})$$

In 2004, Marshall and McClean^19^ discussed the possibility of estimating the probability that an individual will experience the event of interest from each transient state, denoted *π_k_*, which is obtained by considering the probability that an individual first survives until the *k^th^* phase and then gets absorbed. A generalised expression for this probability is given by

(11) $$\pi_{k}=(\frac{\mu_{k}}{\mu_{k}+\lambda_{k}})\overset{k-1}{\underset{j=1}{\Pi }}(\frac{\lambda_{j}}{\mu_{j}+\lambda_{j}}),k=1,\ldots ,n$$

where $\lambda_{n}=0$.

According to the state from which they leave the system, individuals can then be categorised in the ratio $\pi_{1}:\pi_{2}:\ldots :\pi_{n}$ making it possible to determine the lower and upper bounds of the time spent in each state, *S_g_*, by the following equation

(12) $$S_{g}=\{t^{\left(j\right)}:M\sum_{k=1}^{g-1}\pi_{k}<j\leq M\sum_{k=1}^{g}\pi_{k}\},\text{ for }g=1,\ldots ,n$$

where $t^{\left(1\right)},\ldots ,t^{\left(M\right)}$ are the ordered absorption times of the *M* individuals. This allows the identification of which individuals leave the system from which states, subdividing the data into groups with similar survival distributions. Further study of these groups can potentially provide more insight into what characteristics influence how individuals move through the system. This approach, however, imposes the assumption that all individuals who are absorbed from the first phase do so before any individuals are absorbed from the second phase, and so on. It does not allow for a scenario whereby one individual may quickly deteriorate through the system and absorb from the final phase faster than another individual may absorb from the first phase, for example.

### 3.3 Fitting procedure

Maximum likelihood estimation is the most common approach taken to fit the Coxian phase-type distribution, where the log likelihood function is given by

(13) $$\log L=\sum_{i=1}^{M}\log f(t_{i})=\sum_{i=1}^{M}\log(p\exp\{Qt_{i}\}q)$$

Employing the forward Kolmogorov equation^20^ to calculate the matrix exponential of the probability density function (7), whilst utilising the probabilities of absorption from each state, allows an analytic expression for the probability density function to be derived,^21^ as shown below, speeding up the fitting process

(14) $$f(t_{i})=\sum_{h=1}^{n}\pi_{h}(\sum_{k=1}^{h}C_{\mathrm{kh}}(\lambda_{k}+\mu_{k})e^{-(\lambda_{k}+\mu_{k})t_{i}})$$

where

(15) $$\pi_{h}=\frac{\mu_{h}}{\mu_{h}+\lambda_{h}}\overset{h-1}{\underset{j=1}{\Pi }}(\frac{\lambda_{j}}{\mu_{j}+\lambda_{j}})$$

(16) Ckh=Πj=1j≠kh(λj+μjλj+μj-(λk+μk))

and where $\lambda_{n}=0$.

Faddy and McClean,^17^ Faddy^22^ and Marshall and McClean^23^ each employed the Nelder–Mead^24^ simplex algorithm to maximise the likelihood, making use of inbuilt MATLAB®^25^ optimisation functions, for example, fminsearch. Asmussen et al.^26^ described a general method of employing the EM algorithm to estimate the parameters of phase-type distributions by treating the distribution as a multistate model problem with missingness; such an approach is computationally more intensive than the Nelder–Mead. Olsson^27^ extended this methodology to include right-censored and interval-censored data.

Alternative approaches, which are used much less commonly, include moment matching techniques^28,29^ and least squares utilising a quasi-Newton minimisation algorithm.^30,31^ Marshall and Zenga^32,33^ and Payne et al.^34^ have previously discussed various fitting procedures, and their efficiency, in more detail.

In order to identify the optimal number of phases which best describes the distribution of the data, it is necessary to sequentially fit an increasing number of phases, starting with *n* = 1 (i.e. an exponential distribution), until the improvement of the fit becomes negligible. This is determined by looking at the Akaike information criterion (AIC), Bayesian information criterion and by conducting likelihood ratio tests (LRTs).

A common problem encountered when fitting the Coxian phase-type distribution is that the fit is very strongly influenced by the initial parameter values.^35^ Consequently, to ensure that the best fit has been achieved, an iterative approach is taken whereby different initial values are chosen for each iteration and the best fit is informed by the log-likelihood.

## 4 Coxian phase-type regression model

As it is possible to make inferences from the parameters of the Coxian phase-type distribution on individuals’ rates of flow through the system, a logical progression is to consider the incorporation of covariates into the model so as to evaluate their effect on these rates of flow.

Various approaches of incorporating covariates have previously been explored. Faddy et al.^36^ considered evaluating covariate dependence through a generalised linear model whereby the mean length of stay within the system is given by the log-linear regression exp{a+xα'}, where **x** is a vector of covariate values with corresponding regression parameters α, estimated by maximum likelihood. McGrory et al.^37^ explored a fully Bayesian approach to this covariate dependent mean. Similarly, Faddy and McClean^38^ and McClean et al.^39^ incorporated covariates in accordance with a generalised linear model framework whereas Marshall and McClean,^23^ alternatively, conditioned on covariates when fitting phase-type distributions so as to identify cohorts of individuals with similar survival distributions.

Tang et al.,^14^ in 2012, described a Coxian phase-type regression model within which the rate parameters of the probability density function, equation (7), are replaced with λk=λ0kexp{-xi'α} and μk=μ0kexp{-xi'α}, where $x_{i}$ is a vector of covariate values for individual *i* with corresponding parameters given by α and $\lambda_{0k}$ and $\mu_{0k}$ represent the transition rates of a baseline individual. Thus, the probability density function is given by

(17) f(t)=pexp{exp{-xi'α}Qt}(exp{-xi'α}q)=pexp{Q~t}q~

where

(18) Q~=exp{-xi'α}Q

(19) q~=exp{-xi'α}q

and where **Q** and **q** are defined as before. The significance of the parameters relating to the covariate effects is validated by using bootstrapping techniques. Within this model set-up, it is assumed that the covariates effect is constant across all transition rates within the system. Previous research has successfully used this approach to evaluate the impact of various covariates on the length of stay of geriatric patients in hospital.^40^

Within this paper, the predicted random effects, estimated in Stage 1 using a LME model by equation (4), shall be incorporated within the Coxian phase-type regression model in Stage 2, where their effect on the rate parameters shall be quantified. The probability density function is thus given by

(20) f(t)=pexp{exp{-bi'α}Qt}(exp{-bi'α}q)

where $b_{i}$ are the predicted random effects, as defined before.

## 5 Application to CKD

CKD is a degenerative condition whereby an individual’s kidney function gradually reduces over time, culminating in renal failure whereby dialysis treatment and a kidney transplant are necessary.^41^ The rate at which this deterioration occurs can vary significantly amongst individuals and, consequently, different treatment interventions are necessary.

It has been estimated by a National Health Service (NHS) Kidney Care Report^42^ that there are approximately 1.8 million people currently diagnosed with CKD in England, with a further one million people undiagnosed, costing the NHS £1.45 billion in 2009/2010. Furthermore, the number of CKD cases is increasing, with the cost to the NHS in the UK more than doubling from £445 million in 2002/2003, making CKD a prevailing challenge for healthcare providers.^43^ The Kidney Care Report also noted that 95% of this expenditure was attributed to secondary care of CKD patients, in particular the costs of renal replacement therapies, such as dialysis. Consequently, it is mutually beneficial to both patients and healthcare providers to more accurately model the behaviour of CKD so as to provide treatment interventions with greater accuracy in a more cost-efficient manner.

It is commonly observed that anaemia, a condition where the body has a reduced volume of red blood cells, occurs concurrently with CKD and that both conditions deteriorate at a similar rate.^44^ Consequently, haemoglobin (Hb) levels, a protein found in red blood cells which is responsible for the transport of oxygen around the body, is seen as an emerging CKD biomarker.

This section utilises the new two-stage approach in the analysis of data collected from various renal centres across Northern Ireland by the NI Renal Information Service from April 2002 until December 2011 and contains multiple repeated measures on the Hb levels of 577 individuals undergoing haemodialysis, along with various additional covariates relating to their health state. The average number of observations per individual is 18 with a maximum of 84 and minimum of 2 and the average age when starting haemodialysis is 54 years old, with a minimum of 9.73 and maximum of 78.73.

**Figure 2.**
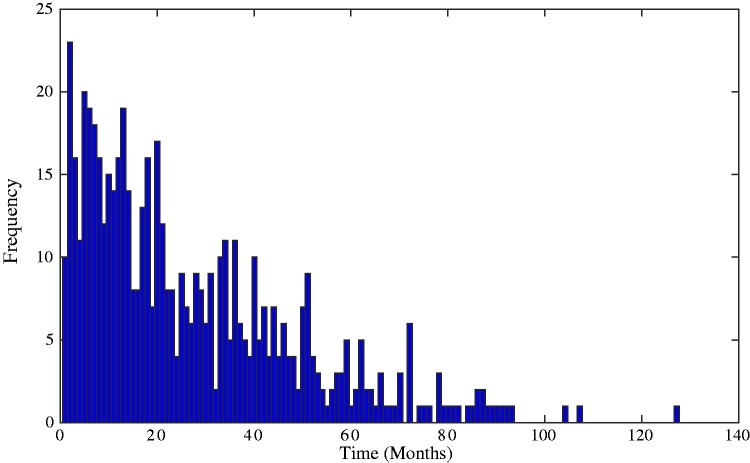
Histogram showing the distribution of death times of individuals suffering from CKD. The observed positive skew, which is typical of survival data, can be well represented by the positively skewed Coxian phase-type distribution. CKD: chronic kidney disease.

The distribution of the observed death times of these 577 individuals, displayed in Figure 2, resembles a typical survival distribution which the Coxian phase-type distribution has been shown in previous research to suitably represent.

The model fitting procedure is described in the following sections.

### 5.1 Stage 1: LME model

In Stage 1, a LME model is employed to analyse individuals’ haemoglobin levels, where the following observed covariates were found to have a significant influence and thus were incorporated as fixed effects within the model:

- Observation time, recorded in months from the time an individual started haemodialysis,
- Age at commencement of haemodialysis,
- Mean corpuscular volume and mean corpuscular haemoglobin concentration, two kinds of red blood cell indices giving measures of the average red blood cell size and the amount of haemoglobin relative to the size of the cell, respectively. Both are potential biomarkers not analysed readily in current renal research.
- Ferritin, a protein responsible for the storing and release of iron in the body,
- Creatinine, a breakdown product produced by the body’s muscles which is filtered from the blood by the kidneys; it is often studied as a common indicator of renal health,
- Urea, a waste product produced by the body which is also filtered from the blood by the kidneys,
- Erythropoietin (EPO) treatments (Aranesp, Epoetin Alfa, Epoetin Beta and Other): EPO is a hormone which controls red blood cell production. Commonly, individuals suffering from CKD and anaemia are given EPO treatments to increase the production of red blood cells,
- Iron treatments (Iron Hydroxide, Venofer and No Iron), given to treat iron deficiency in CKD or anaemic patients.

Furthermore, individuals were also allowed to deviate from the population average in terms of their intercept and rate of change over time by the inclusion of two random effects within the model. The significance of the random effects was confirmed using LRTs to compare the nested models, as shown in Table 1.

**Table 1.** Likelihood ratio tests showing the significance of the random intercept and random slope.

	−$2^{*}$LogLik	Df
Model 1: Ordinary linear model	37,520.90	14
Model 2: Random intercept model	35,365.46	15
Model 3: Random intercept and slope model	34,955.76	17
Compare:	Chi Sq.	p-value
Test 1: Model 1 and Model 2	2155.40	<0.001
Test 2: Model 2 and Model 3	409.71	<0.001

Df: Degrees of freedom; LogLik: Log-likelihood.

Therefore, the LME model with a random intercept and slope (Model 3) was chosen and is given by

(21) $$\begin{array}{ll} Hb_{ij}= & \;{\text{Intercept}}_{i}\beta_{0}+{\text{Time}}_{ij}\beta_{1}+{\text{Age}}_{ij}\beta_{2}+{\text{MCV}}_{ij}\beta_{3}+{\text{MCHC}}_{ij}\beta_{4}+{\text{Ferritin}}_{ij}\beta_{5}\\ & +{\text{Creatinine}}_{ij}\beta_{6}+{\text{Urea}}_{ij}\beta_{7}+{\text{Aranesp}}_{ij}\beta_{8}+{\text{EpoetinAlfa}}_{ij}\beta_{9}+{\text{OtherEPO}}_{ij}\beta_{10}\\ & +{\text{IronHydroxide}}_{ij}\beta_{11}+{\text{Venofer}}_{ij}\beta_{12}+{\text{Intercept}}_{i}b_{i0}+{\text{Time}}_{ij}b_{i1}+\epsilon_{ij} \end{array}$$

Note that ‘Epoetin Beta’ and ‘No Iron’ are the baselines for EPO and Iron treatment, respectively, and the remaining (continuous) variables are centred around their means. The various fixed effects, along with their corresponding parameter estimates, are given in Table 2, along with the variance and covariance of the random effects. It can be observed from their corresponding p-values that all covariates have a significant impact on individuals’ haemoglobin levels.

**Table 2.** Parameter estimates of the fixed effects of the CKD model.

Fixed effects		Estimate	Standard error	p-value
(Intercept)		11.322	0.069	<0.001
Time (months)		−0.007	0.002	0.003
Age		0.017	0.004	<0.001
MCV		0.027	0.003	<0.001
MCHC		0.289	0.015	<0.001
Ferritin		<0.001	<0.001	<0.001
Creatinine		0.002	<0.001	<0.001
Urea		0.019	0.004	<0.001
EPO treatment:	Aranesp	0.028	0.058	0.635
*(Baseline=Epoetin Beta)*	Epoetin Alfa	−0.244	0.101	0.016
	Other	−1.544	0.565	0.006
Iron treatment:	Iron Hydroxide	0.250	0.055	<0.001
*(Baseline = No Iron)*	Venofer	0.248	0.033	<0.001
Random effects			Variance	Covariance
(intercept)			1.379	
Time (months)			0.002	−0.033
Residual error			1.313	

CKD: chronic kidney disease; EPO: erythropoietin; MCHC: mean corpuscular haemoglobin concentration; MCV: Mean corpuscular volume.

**Figure 3.**
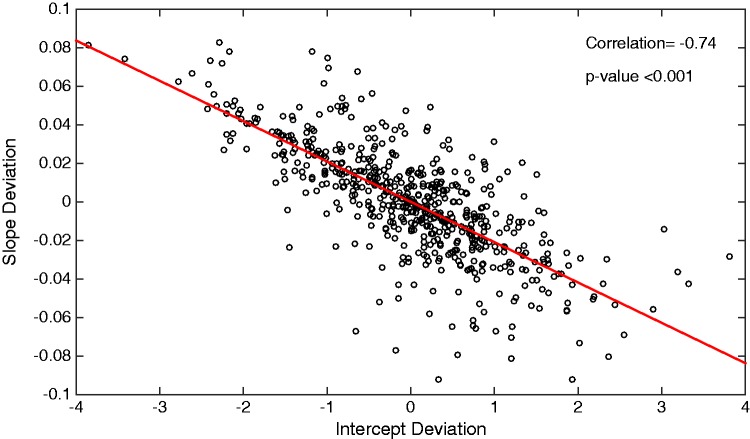
Scatter plot showing the high negative correlation (−0.74) between individuals’ deviations from the population-average haemoglobin slope and intercept; individuals with a higher than average intercept deviation typically have a smaller than average slope deviation.

From the predicted random effects it is possible to obtain, for each individual, a measure of how their Hb level deviates from the population-average intercept, $b_{i0}$, and slope, $b_{i1}$. Plotting each individual’s random intercept against their random slope shows the negative correlation observed between the random effects. Individuals with a smaller than average initial Hb level tend to have a steeper slope and thus a greater change in Hb over time, whereas individuals with a greater than average initial Hb level have a shallower slope, as can be seen in Figure 3. Such trends are in accordance with those observed in previous CKD literature where, for example, Gilbertson et al.,^45^ observed greater variations over time amongst haemodialysis patients with lower initial Hb levels.

### 5.2 Stage 2: Coxian phase-type regression model

In Stage 2, a Coxian phase-type regression model is used to determine the underlying states of CKD and to estimate the rates of deterioration through them. Individuals’ deviations from the population-average Hb levels, the predicted random effects, are incorporated as covariates within the Coxian phase-type regression model so as to evaluate their effect on these rates of flow.

The probability density function of the model, representing individuals who die within the observation period, is given by

(22) $$f(t_{i})=p\exp\{\exp\{-b_{i0}\alpha_{1}-(b_{i1}t_{i})\alpha_{2}\}Qt_{i}\}(\exp\{-b_{i0}\alpha_{1}-(b_{i1}t_{i})\alpha_{2}\}q)$$

where *t_i_* is the event time for individual *i* and $b_{i0}$ and $b_{i1}$ are the predicted random effects defined previously.

The estimated parameters of the Coxian phase-type regression model, along with the corresponding log-likelihood and AIC values are given in Table 3.

**Table 3.** Table showing parameter estimates, along with corresponding AIC values, of the fitted Coxian phase-type regression models.

Phase	Parameter	Estimate	Log likelihood	AIC	LRT $\chi^{2}$ (p-value)
1	μ^1	0.038	−2466.368	4938.737	–
	α^1	0.253			
	α^2	0.162			
2	μ^1	$1.43\times 10^{-14}$	−2449.229	4908.457	34.278 (<0.0001)
	μ^2	0.425			
	λ^1	0.041			
	α^1	0.245			
	α^2	0.158			
3^a^	μ^1	$9.02\times 10^{-11}$	−2443.927	4901.855	10.604 (0.002)
	μ^2	0.435			
	μ^3	0.060			
	λ^1	0.060			
	λ^2	0.467			
	α^1	0.246			
	α^2	0.156			
4	μ^1	$2.99\times 10^{-7}$	−2442.342	4902.685	3.170 (0.102)
	μ^2	0.238			
	μ^3	0.000			
	μ^4	0.077			
	λ^1	0.086			
	λ^2	0.230			
	λ^3	0.077			
	α^1	0.228			
	α^2	0.140			

AIC: Akaike information criterion; LRT: likelihood ratio test.

a Indicates the optimal number of phases to fit to the data.

**Figure 4.**
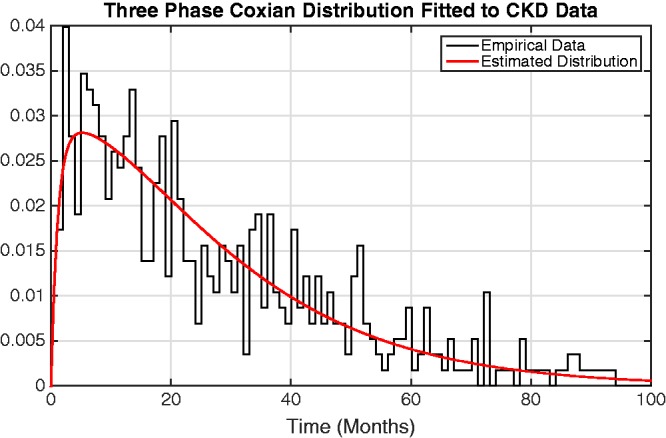
Graph showing the probability density function of the estimated Coxian phase-type distribution overlaid upon the time to death (in months) from the empirical CKD data. CKD: chronic kidney disease.

A LRT was also conducted, comparing a fit of *n* phases with that of *n* − 1 phases to evaluate if there exists a significant contribution from the additional parameters. It can be observed from the corresponding p-values of the LRT that a three-phase Coxian phase-type regression model provides the optimal fit to the data. Plotting the distribution described by the rate parameters, denoted *μ_k_* and *λ_k_*, indicates that it provides a suitable fit to the data, as shown in Figure 4.

Looking at the covariate parameters, denoted by *α*_1_ and *α*_2_, it can be interpreted that a one unit increase in an individual’s initial Hb level results in a multiplicative rate-of-flow increase of $\exp\{-\alpha_{1}\}=\exp\{-0.246\}=0.781$. Similarly, a one unit increase in an individual’s rate of change over time results in a multiplicative rate-of-flow increase given by $\exp\{-\alpha_{2}t\}=\exp\{0.156\times 1\}=0.856$ per month after commencing haemodialysis, compared to that of the population average. The baseline in such a model ($b_{i0}=b_{i1}=0$) represents an individual whose haemoglobin level is equal to the population average.

For example, individual 143 has an intercept deviation of −3.850 and a slope deviation of 0.081, observed at time 6.72 months. Therefore, it can be calculated that this individual is going to progress through the system at a rate of flow which is exp{-(-3.850×0.246})-((0.081×6.72)×0.156)}=2.368 times that of the population average.

**Figure 5.**
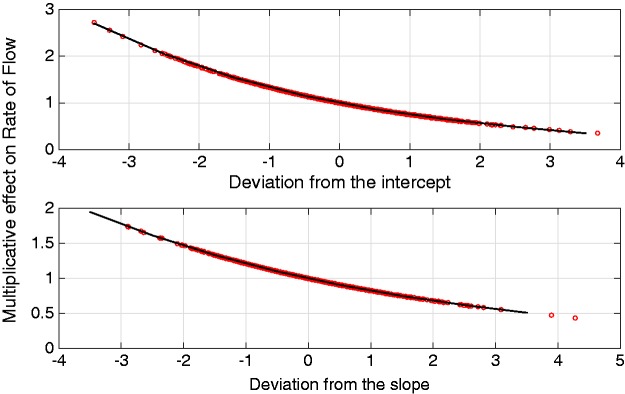
Graph showing the effect of individuals’ deviations from the population-average intercept and slope on their rates of flow through the Coxian phase-type distribution. For example, individuals whose intercept deviation (i.e. initial Hb level) is 3 units below the population-average (−3 along the x-axis) transition through the system ∼2.3 times faster than the population average, read from the y-axis.

Figure 5 provides a graphical representation of the effect of an individual’s deviation from the population average on their rate of flow through the system. From this figure, it can be observed that individuals with a lower than average initial Hb level and change in Hb over time transition faster through the system while those with a higher than average initial Hb level and change in Hb over time transition more slowly. This agrees with previous CKD research by McCrink et al.^46^ who identified that individuals with higher than average initial haemoglobin levels typically had better survival, but goes further by allowing the underlying disease stages to be identified and for inferences of the rates of flow through them to be made.

Finally, bootstrapping techniques were used to estimate standard errors of the covariate parameter estimates α^1 and α^2, which were then used to test the significance of the parameters.

The 95% confidence intervals (CI) can also be calculated, as shown

(23) $$e^{-\alpha_{i}}\pm 1.96\text{ s}.\text{e }\{e^{-\alpha_{i}}\}$$

If the value 1 lies within this interval then the parameter *α_i_* does not have a significant effect on the rates of flow. The CIs for the intercept and slope deviations are given by

(24) $$\begin{array}{ll} & e^{-\alpha_{1}}\pm 1.96\text{ s}.\text{e }\{e^{-\alpha_{1}}\}=e^{-0.246}\pm (1.96^{*}0.0613)=(0.662,0.902)\\ & e^{-\alpha_{2}}\pm 1.96\text{ s}.\text{e }\{e^{-\alpha_{2}}\}=e^{-0.156}\pm (1.96^{*}0.0463)=(0.764,0.946) \end{array}$$

and hence it can be concluded that both covariates have a significant effect on rate of flow.

Utilising the parameter estimates of the optimal three-phase Coxian phase-type distribution, it is possible to estimate the probability of experiencing the event of interest, in this case death, from each phase, using equation (11), as shown

(25) $$\begin{array}{l} \pi_{1}=\left(\frac{\mu_{1}}{\mu_{1}+\lambda_{1}}\right)=\left(\frac{9.02\times 10^{-11}}{9.02\times 10^{-11}+0.060}\right)=1.50\times 10^{-9}\\ \pi_{2}=\left(\frac{\mu_{2}}{\mu_{2}+\lambda_{2}}\right)\left(\frac{\lambda_{1}}{\mu_{1}+\lambda_{1}}\right)=\left(\frac{0.435}{0.435+0.467}\right)\left(\frac{0.060}{0.000+0.060}\right)=0.482\\ \pi_{3}=\left(\frac{\lambda_{2}}{\mu_{2}+\lambda_{2}}\right)\left(\frac{\lambda_{1}}{\mu_{1}+\lambda_{1}}\right)=\left(\frac{0.467}{0.435+0.467}\right)\left(\frac{0.060}{0.000+0.060}\right)=0.518 \end{array}$$

In order to determine which individuals die from which phase, the ordered event times are split in the ratio $\pi_{1}:\pi_{2}:\pi_{2}$ and the upper and lower bounds of each phase can be determined, using equation (12), as shown

(26) $$\begin{array}{l} S_{1}=0\\ S_{2}=\{t^{\left(j\right)}:0<j\leq 278\}\\ S_{3}=\{t^{\left(j\right)}:278<j\leq 577\} \end{array}$$

Thus, it can be interpreted that approximately 48% of deaths occurred from phase two of the model and the remaining 52% from phase three, with only a small chance of an individual dying from the first phase. Considering these phases to represent distinct stages of CKD progression, it could be inferred that those individuals who die from phase two make up a sicker cohort of patients, compared to those who die from phase three, as they have worse survival.

By inspecting the data, it can be observed that by time point 19.68 months, all individuals have moved through phase two of the system into either the absorbing phase or phase three. Further analysis of those individuals who die from each phase may make it possible to identify factors which influence individuals’ death times. Forecasting such information allows different treatment plans to be prescribed depending on the expected rate of deterioration of an individual though the system.

### 5.3 Individual-specific survival and hazard estimates

By looking at the parameters of the fitted Coxian phase-type regression model, the probability of survival for some future time point, *t*, can be estimated at both a population level (as in previous literature) and, by utilising the proposed two-stage approach, at an individual level, providing dynamic individual-specific survival probabilities. These survival probabilities are estimated using the survivor function of the Coxian phase-type distribution, given by

(27) S(t)=pexp{Qt}1

where 1 is a vector of 1 s and **Q** and **p** are as defined in equations (8) and (9), respectively. This can be written analytically as shown

(28) $$S(t)=\sum_{h=1}^{n}\pi_{h}(\sum_{k=1}^{h}C_{\mathrm{kh}}(\lambda_{k}+\mu_{k})e^{-(\lambda_{k}+\mu_{k})t})$$

where

(29) $$\pi_{h}=\frac{1}{\mu_{h}+\lambda_{h}}\overset{h-1}{\underset{j=1}{\Pi }}(\frac{\lambda_{j}}{\mu_{j}+\lambda_{j}})$$

(30) Ckh=Πj=1j≠kh(λj+μjλj+μj-(λk+μk))

and where $\lambda_{n}=0$.

**Figure 6.**
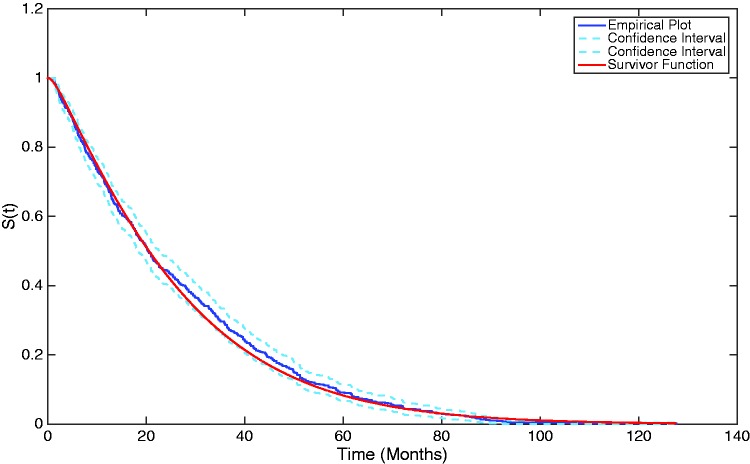
The population-average survivor function from the estimated Coxian phase-type distribution overlaid upon the empirical survivor plot.

Therefore, the population-average survival probability of a baseline individual through time can be calculated and plotted, as shown in Figure 6. The estimated survivor function is overlaid upon the empirical survival probability, highlighting that the three-phase Coxian phase-type regression model utilised in this work provides a suitable estimate to the survival probability.

**Figure 7.**
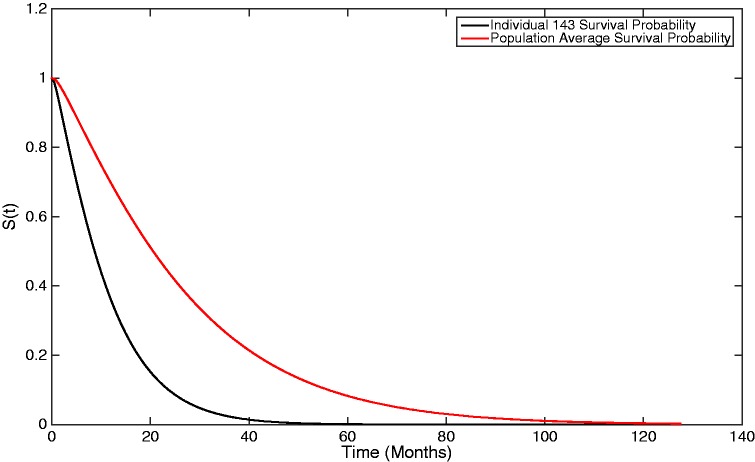
A graph showing both the population-average and an individual-specific survivor plot, generated from the estimated Coxian phase-type distribution.

By incorporating the individual-specific covariate effects, it is further possible to estimate personalised survival probabilities utilising the two-stage approach proposed in this research, where the individualised rates of flow are given instead by $\mu_{k}^{*}\exp\{-0.246^{*}b_{i0}-0.156^{*}b_{i1}t\}$ and $\lambda_{k}^{*}\exp\{-0.246^{*}b_{i0}-0.156^{*}b_{i1}t\}$. For instance, looking again at individual 143, the parameters which describe this individual’s rates of flow are given by $2.368\mu_{k}$ and $2.368\lambda_{k}$ for movement out of and through the system, respectively. Therefore, this individual is expected to progress through their illness, and the different stages of CKD, at a faster rate than the population average, thus making them a candidate for referral to a specialist for a more personalised treatment plan. In such consultations, the use of Figure 7, the survival probability through time for this individual, along with the population-average survival probability, would prove extremely useful. Such a plot is dynamic in nature where, as new longitudinal information is received from a patient over time, it updates, allowing more accurate and representative survival information to be utilised by the clinician and relayed to the patient.

It can be observed that, as this individual transitions through the process, on average, 2.368 times faster than a baseline individual, their survival probability is smaller, i.e. they accelerate towards death faster.

It is also possible to estimate both population-average and individual-specific hazards, where the hazard function is given by

(31) $$h(t)=\frac{f(t)}{S(t)}=\frac{p\exp\{Qt\}q}{p\exp\{Qt\}1}$$

**Figure 8.**
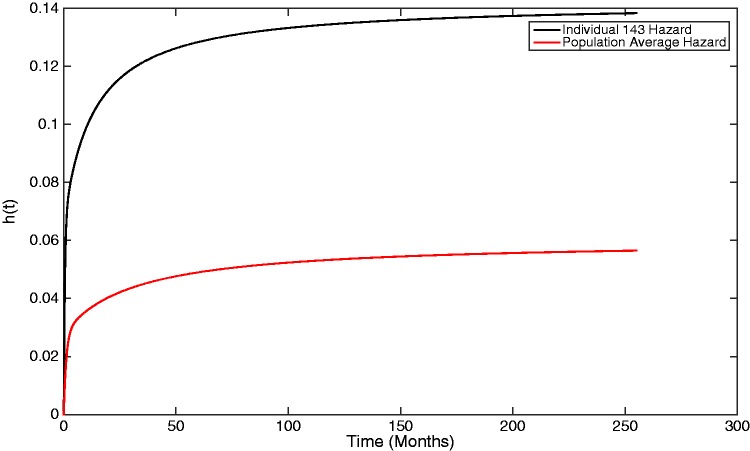
A graph showing both the population-average and an individual-specific hazard plot, generated from the estimated Coxian phase-type distribution.

For an exponential distribution, i.e. a one-phase Coxian, the hazard is constant and equal to the absorption parameter of the distribution. For a Coxian phase-type distribution with multiple phases, the hazard is constant within each phase, and equal to the absorption rate of that phase, *μ_k_*, but can vary between phases. The problem, however, is that it is not possible to know which phase an individual belongs to at each time point, *t*. Therefore, the hazard will be weighted based on the probability of which state an individual will belong to. The overall hazard does, however, converge towards the hazard of the final state as, over time, individuals are increasingly likely to belong to this state. Figure 8 shows the baseline hazard representing the population average, which converges towards the fixed hazard of the final phase, $\mu_{3}=0.06$. Again, the key benefit of the proposed two-stage model is that it allows individual-specific hazards to be plotted. The hazard for individual 143 is also given within Figure 8 and can be observed to converge towards the individual’s personalised hazard in the third phase, $\mu_{3}=0.06^{*}2.368=0.142$.

Individual 143 has an increased hazard of experiencing the event of interest compared to what would be assumed if a population-average approach was used to make inferences about the individuals within the study. By instead using the individual-specific plots, made possible by the two-stage approach, high-risk individuals can be identified and recommended for alternative treatment.

Similarly, the population-average cumulative hazard can also be plotted, utilising the *μ_k_* and *λ_k_* parameters, as before. The cumulative hazard is given by

**Figure 9.**
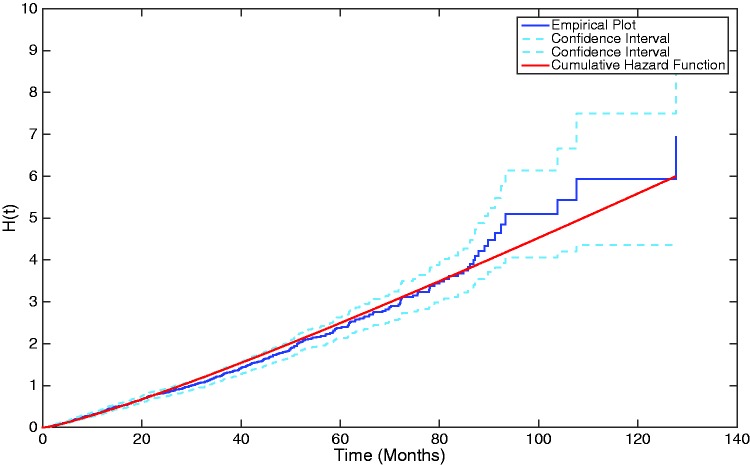
A plot showing the cumulative hazard function generated from the estimated Coxian phase-type distribution overlaid upon the empirical plot of the cumulative hazard.

(32) H(t)=-log(S(t))=-log(pexp{Qt}1)

and is shown in Figure 9, overlaid upon the empirical plot, further illustrating how well the three-phase Coxian phase-type distribution fits to the data.

## 6 Conclusions and further work

This paper introduces a two-stage approach to the joint analysis of longitudinal response and survival outcome utilising a LME model and a Coxian phase-type regression model, respectively. Such an approach allows the effect of individual deviations from a population-average longitudinal response on survival and disease progression to be identified. What’s more, by employing the Coxian phase-type distribution to represent the survival process in place of the standard Cox PH model, found commonly in the literature, additional information pertaining to rates of deterioration through sequential disease stages (the Coxian phases) was also obtained, providing further insight into how the disease under investigation will progress. To the author’s knowledge, no previous research has incorporated a Coxian regression model into this type of analysis. In addition to this, it has been shown how such a two-stage model may be utilised to produce personalised rates of flow, along with personalised survival and hazard plots, as illustrated, thus allowing more sophisticated, individualised treatment plans to be developed for each patient – an invaluable technique for personalised medicine.^47^

Within more recent joint modelling literature, where the survival process is represented by the Cox PH model, the parameters of the longitudinal and survival processes are estimated simultaneously through a single joint likelihood. The purpose of this is to reduce the possible bias introduced to the estimates of the longitudinal parameters and random effects due to informative dropout. For instance, individuals who die earlier within the observation period typically do not have as many repeated measures collected on their longitudinal response, meaning they can become under-represented within the sample. Extensions of the work presented will investigate a similar joint likelihood approach to the methods discussed within this paper, using the Coxian phase-type distribution to represent the survival process.
